# Right-to-Left Shunt Does Not Increase the Incidence of Silent Lacunar Infarcts in Patients with Migraine

**DOI:** 10.1155/2015/749745

**Published:** 2015-07-21

**Authors:** Wei Du, Xiujuan Wu, Yingqi Xing, Yunlong Geng, Jing Bai, Xiaonan Song

**Affiliations:** ^1^Neuroscience Center, Department of Neurology, The First Hospital of Jilin University, Jilin University, Changchun 130021, China; ^2^Department of Clinical Medicine, Jilin University, Changchun, Jilin 130021, China

## Abstract

Right-to-left shunt (RLS) is associated with cryptogenic stroke and migraine. Herein we investigated the relationship between RLS and silent lacunar infarcts in patients with migraine. A total of 263 patients with migraine who met eligibility criteria were enrolled from January 2010 to December 2011, among which 127 subjects fell into RLS group. Baseline demographics were comparable between RLS and non-RLS groups (*P* > 0.05). The incidence of silent lacunar infarcts in RLS group was not significantly different from that of the non-RLS group (25.2% versus 21.3%, *P* > 0.05). Furthermore, we found that the incidence of silent lacunar infarcts in permanent and latent RLS subgroups was comparable with non-shunt RLS subgroup (28.6% versus 24% versus 21.3%, *P* > 0.05). Similarly, the incidence of silent lacunar infarcts in the non-RLS group, mild-shunt group, and large-shunt group was also comparable (21.3% versus 23.8% versus 29.3%, *P* > 0.05). In addition, RLS did not increase the incidence of silent lacunar infarcts in migraine patients with elder age (<50 years age group: 15.8% versus 17.9%; ≥50 years age group: 53.1% versus 37.5%, both *P* > 0.05). In conclusion, RLS does not increase the incidence of silent lacunar infarcts in patients with migraine. Further prospective studies are warranted to validate this finding.

## 1. Introduction

Silent lacunar infarcts can eventually cause cognitive dysfunction, dementia, and depression [1]. Additionally, patients who have these infarcts have been reported to have a significantly increased risk of symptomatic stroke, which reduces the patient's quality of life and ability to work [2]. With improvements in medical technology and the accuracy of diagnostic instruments, silent lacunar infarcts can now be more precisely diagnosed; however, their etiology and pathogenesis remain unclear.

In recent years, studies have focused on whether the right-to-left shunt (RLS) leads to an increased risk of stroke and the conclusions have been controversial. To our knowledge, the most common cause of RLS is a patent foramen ovale (PFO), which was not previously thought to cause or trigger reversed blood flow affecting the hemodynamics of the heart. However, recent studies have shown that PFO is related to unexplained cerebral infarction, migraine, decompression sickness, systemic arterial embolism, and sleep apnea. Several studies have shown that a significant statistical relationship exists between RLS and migraine [3–5]. In addition, RLS is widely recognized as causing a “paradoxical embolism” type stroke, which refers to emboli from the venous system and/or right atrium flowing into the systemic circulation, leading to ischemic stroke and embolism of the heart, kidney, and peripheral arteries. Controversy exists over the mechanism of paradoxical embolism, but a potential direct relationship has been established between stroke and PFO. In a previous study [6], symptomatic stroke with acute infarcts revealed by magnetic resonance imaging (MRI) scans was found to be associated with PFO. However, few studies have focused on whether silent lacunar infarcts in patients with migraine correlate with RLS. In this study, we investigated the relationship between RLS and silent lacunar infarcts; that is, whether RLS could increase the prevalence of silent lacunar infarcts in patients with migraine. We also explored the relationship between different types of RLS, detected by the transcranial Doppler (TCD) bubble test, and the prevalence of silent lacunar infarcts, revealed by MRI of the head.

## 2. Subjects and Methods 

### 2.1. Subjects

The study was approved by the ethics committee of The First Hospital of Jilin University, Changchun, China. Written informed consent was obtained from all the recruited patients. From January 2010 to December 2011, patients with migraine who visited the neurology clinic at The First Hospital of Jilin University and simultaneously met our inclusion and exclusion criteria were enrolled in the study. The inclusion criteria were as follows: (1) patients had a history of migraine for at least 1 year, and the diagnosis of migraine met with the diagnostic criteria for migraine revised by the Head and Face Pain Classification Committee of the International Headache Society [7]; (2) patients had no symptoms of transient ischemic attack (TIA) or ischemic stroke and had signed the consent forms. The exclusion criteria were as follows: (1) patients who refused to undergo a head MRI or the TCD bubble test and (2) those with intracranial or extracranial artery stenosis demonstrated by TCD and carotid ultrasound, or with other central nervous system diseases such as multiple sclerosis, cerebral hemorrhage, cerebral vascular malformations, and brain tumors. All the patients enrolled underwent conventional TCD (EMS-9, Delica, China), carotid ultrasound (IU22, Phillips, Andover, MA), head MRI, and TCD bubble test. In addition, the common risk factors for ischemic stroke in these patients, including hypertension, diabetes, coronary heart disease, hyperlipidemia, and history of smoking, were recorded.

### 2.2. TCD Bubble Test

A German DWL Multi-DopX4 transcranial Doppler was used in the TCD bubble test. The head fixed probe was applied, the probe frequency was 2 MHz, and the unilateral middle cerebral artery was monitored. The monitoring depth was 40–60 mm, and the *M* membrane and blood velocity curve were simultaneously indicated. Specifically trained TCD doctors recorded all parameters. Before the test, patients were asked to practice a standardized Valsalva maneuver. An 18-gauge needle was inserted into the cubital vein in the supine position. Contrast agent was prepared using 9 mL isotonic saline solution, 1 mL air, and a drop of the patient's blood that was vigorously mixed between two 10 mL syringes via a 3-way stopcock. After 30 mixing cycles, the contrast agent was injected as a rapid bolus. The first injection was performed during normal respiration (rest) and the second injection was performed 5 s prior to the start of a 10 s Valsalva maneuver. The time interval between injections was 2 min. The strength of the Valsalva maneuver was measured by peak flow velocity along the Doppler curve. The time at which the first microbubble appeared at the middle cerebral artery (MCA) level was noted. The maximum number of microbubble count (MBs) scored in the MCA for each patient, either during normal breathing or after the Valsalva maneuver, was taken as the estimate of the maximum degree of shunt. Based on the MBs detected by the TCD bubble test, the degree of shunt in patients with RLS was identified and patients were subsequently divided into the mild-shunt group (1–10 MBs) and large-shunt group (>10 MBs), while patients without RLS fell into the non-RLS group, that is, nonshunt group [8]. In addition, the type of RLS could be divided into permanent and latent RLS, depending on when the MBs were detected. Permanent RLS was defined when MBs were detected both at baseline and after the Valsalva maneuver, while latent RLS occurred when MBs were only detected after the Valsalva maneuver. Two ultrasound technologists were designated to assess the extent of RLS and the RLS type in all subjects for whom migraine had been diagnosed by a neurologist.

### 2.3. MRI Examination

In this test, a Siemens Avanto 1.5T superconducting MR imaging system and head quadrature coil were used, including T1WI, T2WI, and fluid attenuated inversion recovery (FLAIR) images. The scan parameters were as follows: T1WI used fast spin echo sequence, TR: 550 ms and TE: 8.4 ms; T2WI used fast spin echo sequence, TR: 4500 ms and TE: 85 ms; FLAIR used inversion recovery sequences, TR: 9000 ms, TE: 103 ms, TI: 2500 ms, thickness 6 mm, interval 1.2 mm, matrix 256 × 256, and field of view 230 mm × 230 mm. Two neuroimaging specialists interpreted the MRI results to measure the existence of silent lacunar infarcts using double-blinded methods. We defined asymptomatic lacunar infarcts as hyperintense lesions on T2WI, with corresponding hypointense lesions with a hyperintense rim on FLAIR, located in the basal ganglia, thalamus, internal or external capsule, or brain stem with a diameter <20 mm and not compatible with clinical findings.

### 2.4. Statistical Analysis

The data obtained were analyzed using SPSS 17.0 statistical software. A two-sample *t*-test was applied to compare the difference between patients and controls. The chi-square test was used to compare the risk factors between patients and controls, including gender, hypertension, diabetes, coronary heart disease, hyperlipidemia, and smoking history. A two-tailed *P* value of <0.05 was considered to be statistically significant.

## 3. Results

### 3.1. Baseline Demographics

In total, 263 patients with migraine who simultaneously met our inclusion and exclusion criteria were enrolled in our study during January 2010 to December 2011. Among them, 127 patients fell into RLS group while 136 were in the non-RLS group. The average age of the patients was 39.8 ± 13.4 years in the RLS group and 37.2 ± 13.6 years in the non-RLS group. The male to female ratio was 49 : 78 in RLS group and 62 : 74 in non-RLS group, respectively. The mean age and sex ratio between the two groups were not significantly different (*P* > 0.05). In addition, incidence of the common risk factors of ischemic stroke (hypertension, diabetes, hyperlipidemia, and smoking history) was not found to be statistically significantly different between the RLS and non-RLS groups, shown in Table 1 (*P* > 0.05).

### 3.2. RLS Did Not Increase the Incidence of Silent Lacunar Infarct in Patients with Migraine

Among the 127 migraine patients with RLS, silent lacunar infarcts were identified in 32 patients (25.2%), whereas 29 out of the 136 patients (21.3%) were found to have silent lacunar infarcts in the non-RLS group. The prevalence of silent lacunar infarcts in the RLS group was similar to the non-RLS group (*P* > 0.05), as demonstrated in Table 2. Taken together with the fact that the mean ages, sex ratio, and the common vascular risk factors between RLS group and non-RLS group were comparable, RLS did not significantly increase the incidence of silent lacunar infarcts in patients with migraine.

### 3.3. Incidence of the Silent Lacunar Infarcts in Migraine Patients with RLS Was Similar Regardless of the Type of RLS

**Figure 1:**
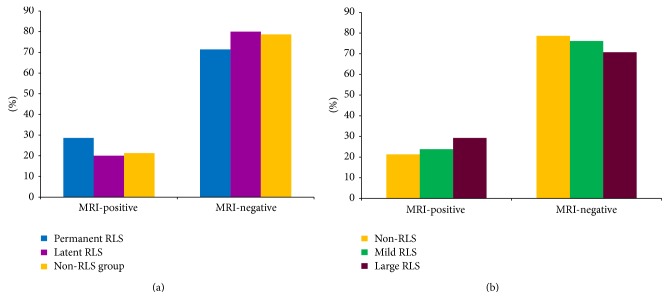
Prevalence of silent lacunar infarcts in patients with migraine and right-to-left shunt (RLS) by different types. Among the 127 patients with RLS, 77 subjects had permanent RLS while 50 had latent RLS. The incidence of silent lacunar infarcts on MRI (MRI-positive) in the permanent RLS and latent RLS subgroups was 22/77 (28.6%) and 10/50 (20%), which was comparable with that in the non-RLS group (*P* > 0.05) (a). Moreover, the incidence of silent lacunar infarcts revealed by MRI in the nonshunt group (non-RLS), mild-shunt group (mild-RLS), and large shunt group (large-RLS) was 21.3%, 23.8%, and 29.3%, respectively, which was comparable (*P* > 0.05) (b).

In addition, the RLS group (127 subjects) could be subdivided into permanent RLS (77 subjects) and latent RLS (50 subjects). The incidence of silent lacunar infarcts seen by MRI in the permanent RLS and latent RLS subgroups was 22/77 (28.6%) and 10/50 (20%) which was comparable with the nonshunt RLS group (28.6% versus 20% versus 21.3%, *P* > 0.05), as demonstrated in Figure 1(a). In addition, according to the count of MBs detected by the TCD bubble test, the RLS group could be subdivided into mild-shunt and large-shunt groups. We further investigated the prevalence of silent lacunar infarcts in the non-RLS group (i.e., nonshunt group), mild-shunt group, and large-shunt group, which was 21.3%, 23.8%, and 29.3%, respectively, shown in Figure 1(b). Although the incidence of silent lacunar infarcts was higher in the large-shunt group; however, it did not reach a significant difference (*P* > 0.05). Collectively, different types of RLS contributed equally to the incidence of the silent lacunar infarcts visible by MRI in patients with migraine.

### 3.4. RLS Did Not Increase the Incidence of Silent Lacunar Infarcts in Older Patients with Migraine

Previous studies have found that the prevalence of silent lacunar infarcts increases with age [9, 10]. All the patients enrolled in this study were divided into one of two groups: ≥50 years or <50 years. They were then subdivided into RLS and non-RLS subgroups based on the existence of RLS in both age groups. We compared the prevalence of silent lacunar infarcts between the RLS and non-RLS subgroups, both for the ≥50 years and the <50 years age groups.  Table 3 shows the prevalence of silent lacunar infarcts in the RLS and non-RLS subgroups, respectively, as well as the ≥50 years and <50 years age groups. We found that the prevalence of silent lacunar infarcts in patients with RLS (i.e., the RLS subgroup) was higher in the ≥50 years age group than the <50 years age group (53.1% versus 15.8%, *P* < 0.05); however, it did not differ from that of the non-RLS subgroup in the ≥50 years age group (53.1% versus 37.5%, *P* > 0.05). Therefore, RLS did not increase the incidence of silent lacunar infarcts in older patients with migraine.

## 4. Discussion

Our previous studies have demonstrated that RLS was associated with cryptogenic stroke. In addition, the compromised dynamic cerebral autoregulation was found in migraine patients with RLS, which might be a potential mechanism of migraine or cryptogenic stroke [11, 12]. In the present study, we further investigated the relationship between silent lacunar infarcts and RLS in patients with migraine and we found that RLS did not increase incidence of silent lacunar infarcts in patients with migraine.

Migraine is a common, chronic, and neurovascular disease induced by multiple factors and characterized by moderate to severe throbbing or pulsating pain, which can be unilateral or bilateral. We previously found that RLS positively associated with migraine [8]. In addition, as shown in a case-controlled study, the prevalence of infarct lesions was significantly increased in patients with migraine [13], together with the fact that latent RLS associated independently with a vertebrobasilar lesion location [14], we proposed the hypothesis that RLS might increase the prevalence of silent lacunar infarcts in patients with migraine.

Previously, it had been found that the provoked RLS was positively associated with ischemic stroke in posterior circulation with incompletely clear mechanism [14]. Wilmshurst and Nightingale supported the hypothesis of a migraine intravenous factor, stating that, under normal circumstances, a number of active substances in venous blood such as 5-HT can be excluded by passage through the lungs; however, when continuous RLS takes place or a Valsalva maneuver is performed, some of these active substances are not filtered by the lungs, leading to RLS-derived molecules that directly enter the systemic circulation and activate platelets or act on the cerebrovascular system to cause migraine [15]. Based on the above hypothesis, RLS, most of which is caused by PFO, is considered to be a channel for paradoxical embolism, which allows active substances or microemboli in vessels to escape from the pulmonary circulation and enter the cerebral circulation, potentially leading to migraine attack or stroke. In addition, it had been found that both migraine and ischemic stroke might be related to abnormal cerebrovascular autoregulation regardless of the existence of RLS [16, 17]. Our previous study had investigated the relationship between dynamic cerebral autoregulation and RLS, and the compromised dynamic cerebral autoregulation was found in migraine patients with RLS, which might be a potential mechanism of migraine or cryptogenic stroke [12]. In the study conducted by Schuchlenz and colleagues, they found that the diameter of the PFO was an independent risk factor for ischemic events in cerebral vessels, especially for recurrent cerebrovascular events [18]. Another study conducted by Anzola and colleagues found that patients with both migraine and stroke had larger shunts than did patients with migraine without stroke, indicating that the larger-shunt RLS might be associated with migraine, as well as increasing the stroke risk in patients with migraine [19]. Similarly, Lamy and colleagues have [20] found that stroke patients with PFO were relatively young with fewer traditional cerebrovascular risk factors, suggesting that PFO might be an independent risk factor for ischemic stroke. Giardini et al. [21] also demonstrated that, in patients with PFO and cryptogenic stroke, a history of migraine and a large spontaneous RLS were related to the high risk of recurrent cerebrovascular events. However, another study [6] has shown that PFO did not increase the risk of stroke and was not an independent risk factor for future cerebrovascular diseases. It is worth noting, however, that not all the patients enrolled in previous studies underwent head MRI examination, which might lead to the missed diagnosis of asymptomatic or silent lacunar infarcts. We postulated that RLS might be associated with the increased incidence of silent lacunar infarcts in patients with migraine which is caused by the compromised dynamic cerebral autoregulation, paradoxical embolism, or the synergistic effect [12, 14]. However, from our current study, we found that RLS did not increase the incidence of silent lacunar infarcts in patients with migraine regardless of the type of the RLS, which still needs further study to validate.

As age is an independent risk factor for cerebrovascular diseases including silent lacunar infarcts, we further investigated the relationship between RLS and silent lacunar infarcts, and we did not find that RLS increased the incidence of silent lacunar infarcts in patients younger than 50 years old, as well as those older than 50 years old. Regardless of the existence of RLS, the prevalence of silent lacunar infarcts in patients with migraine increased with increasing age, suggesting that age plays a pivotal role in the formation of silent lacunar infarcts, which was consistent with previous findings [9, 10].

Nonetheless, there are certain limitations to this study. The TCD bubble test is useful for detecting all meaningful forms of RLS, including heart and pulmonary artery malformations, whereas it cannot locate the position of the RLS. In addition, it can only assess the existence of RLS and the degree of shunting, but it cannot measure the size of the PFO. As the preliminary evidence, the relationship between RLS and silent lacunar infarcts in patients with migraine still warrants further elucidation. Moreover, further studies will be required to investigate the relationship among latent RLS, permanent RLS, and silent lacunar infarcts, as well as the relationship between size of PFO and silent lacunar infarcts in patients with migraine.

In conclusion, RLS does not increase the incidence of silent lacunar infarcts in patients with migraine.

## Figures and Tables

**Table 1:** Comparisons of cerebral vascular disease risk factors between right-to-left shunt (RLS) and non-RLS groups.

Risk factors	RLS group	Non-RLS group	*P* value
	(total 126)	(total 136)	
	*N* (%)	*N* (%)	
Hypertension	7 (6%)	16 (12%)	0.07
Diabetes	1 (1%)	4 (3%)	0.41
Smoking	20 (16%)	24 (17%)	0.68
Coronary heart disease	3 (2%)	3 (2%)	1.00
Hyperlipidemia	4 (3%)	6 (4%)	0.83

**Table 2:** Prevalence of silent lacunar infarcts in the RLS group and non-RLS group.

Feature	RLS group	Non-RLS group
	(total 127)	(total 136)
	*N* (%)	*N* (%)
Existence of silent lacunar infarcts	32 (25.2%)	29 (21.3%)
Absence of silent lacunar infarcts	95 (74.8%)	107 (78.7%)

**Table 3:** Prevalence of silent lacunar infarcts in different age groups.

Groups	MRI findings	RLS subgroup	Non-RLS subgroup
		*N* (%)	*N* (%)
<50-year-old (total 207)	Existence of silent lacunar infarcts	15/95	20/112
		(15.8%)	(17.9%)
	Absence of silent lacunar infarcts	80/95	92/112
		(84.2%)	(82.1%)

≥50-year-old (total 56)	Existence of silent lacunar infarcts	17/32	9/24
		(53.1%)	(37.5%)
	Absence of silent lacunar infarcts	15/32	15/24
		(46.9%)	(62.5%)
